# Multipolar mitosis and aneuploidy after chrysotile treatment: a consequence of abscission failure and cytokinesis regression

**DOI:** 10.18632/oncotarget.6924

**Published:** 2016-01-15

**Authors:** Beatriz Araujo Cortez, Paula Rezende Teixeira, Sambra Redick, Stephen Doxsey, Glaucia Maria Machado-Santelli

**Affiliations:** ^1^ Depto Biologia Celular e do Desenvolvimento, Instituto de Ciências Biomédicas, Universidade de São Paulo, São Paulo, Brasil; ^2^ Depto Genética e Biologia Evolutiva, Instituto de Biociências, Universidade de São Paulo, São Paulo, Brasil; ^3^ Program in Molecular Medicine, University of Massachusetts Medical School, Worcester, MA, USA

**Keywords:** chrysotile, cytokinesis regression, multipolar mitosis, aneuploidy

## Abstract

Chrysotile, like other types of asbestos, has been associated with mesothelioma, lung cancer and asbestosis. However, the cellular abnormalities induced by these fibers involved in cancer development have not been elucidated yet. Previous works show that chrysotile fibers induce features of cancer cells, such as aneuploidy, multinucleation and multipolar mitosis. In the present study, normal and cancer derived human cell lines were treated with chrysotile and the cellular and molecular mechanisms related to generation of aneuploid cells was elucidated. The first alteration observed was cytokinesis regression, the main cause of multinucleated cells formation and centrosome amplification. The multinucleated cells formed after cytokinesis regression were able to progress through cell cycle and generated aneuploid cells after abnormal mitosis. To understand the process of cytokinesis regression, localization of cytokinetic proteins was investigated. It was observed mislocalization of Anillin, Aurora B, Septin 9 and Alix in the intercellular bridge, and no determination of secondary constriction and abscission sites. Fiber treatment also led to overexpression of genes related to cancer, cytokinesis and cell cycle. The results show that chrysotile fibers induce cellular and molecular alterations in normal and tumor cells that have been related to cancer initiation and progression, and that tetraploidization and aneuploid cell formation are striking events after fiber internalization, which could generate a favorable context to cancer development.

## INTRODUCTION

Aneuploidy is characterized by gains and losses of entire chromosomes or parts of chromosomes leading to genomic instability. This phenotype has become a hallmark of solid tumors. The role of aneuploidy in tumorigenesis has been proposed for more than 100 years ago, and it is still under investigation. Aneuploidy can induce or suppress cell proliferation, and thus, promote or inhibit tumor growth [1-4]. Aneuploid cells arise mainly from errors during mitosis such as loss of chromosome cohesion, aberrant microtubule-kinetochore contacts, centrosome amplification and cytokinesis failure. This last dramatic event of cell division failure generates tetraploid cells, which are, in turn, thought to be largely responsible for the aneuploid phenotype [5, 6].

Aneuploidy is also a feature of tumors induced by asbestos *in vivo* [7], and is observed after asbestos treatments *in vitro* [8-10]. These mineral fibers are considered environmental carcinogens and have been implicated in lung cancers and other serious lung diseases (silicosis, carcinomas and mesotheliomas) [11-13], being banished from many countries. There are two groups of asbestos fibers: amphiboles (which include amosite and crocidolite fibers, strongly correlated to lung cancer and mesotheliomas) and serpentines. Serpentines are represented by chrysotile fibers, the only type of asbestos fiber that is still used commercially in many developing countries. Chrysotile is considered more flexible, with a small transverse section and fail to accumulate in the lungs after fiber fragmentation into short pieces. Despite the differences between amphibole and serpentine fibers, both are considered carcinogenic to humans.

Chrysotile fibers when taken up by cells can affect cell morphology and led to mitotic dysfunction. However, little is known about the molecular mechanisms of chrysotile carcinogenesis. Once internalized [9, 14-16], chrysotile fibers can bind proteins, RNAs and organelles and can influence cell behavior [9, 17-20]. For example, they can generate oxygen reactive species that induce cell stress, oxidation reactions, DNA strand breaks and cell death [21-24].

Chrysotile fibers interfere with mitosis leading to mitotic failure, tetraploidization, multinucleation, centrosome amplification and multipolar spindles that generate multiple progeny [8, 9, 25, 26]. However, the mechanisms responsible for aberrant mitotic events have yet to be elucidated [25].

Our earlier work described some of the effects of chrysotile on cancer cells [8], revealing the presence of aneuploidy and multipolar mitosis. In the present study, we focus on the molecular mechanisms underlying these effects, investigating the causes of mitotic and cytokinetic abnormalities and then understanding how aneuploid cells are generated after chrysotile exposure. Also, besides human lung cancer cells we have used a normal epithelial cell culture model to mimic the cells that chrysotile would first encounter when introduced into the lung. This enables us to identify phenotypes, cellular and molecular changes that accompany the tumorigenic process in cells similar to those that become cancerous *in vivo*.

## RESULTS

### Chrysotile treatment induces multinucleation and multipolar divisions in normal and cancer cells

**Figure 1:**
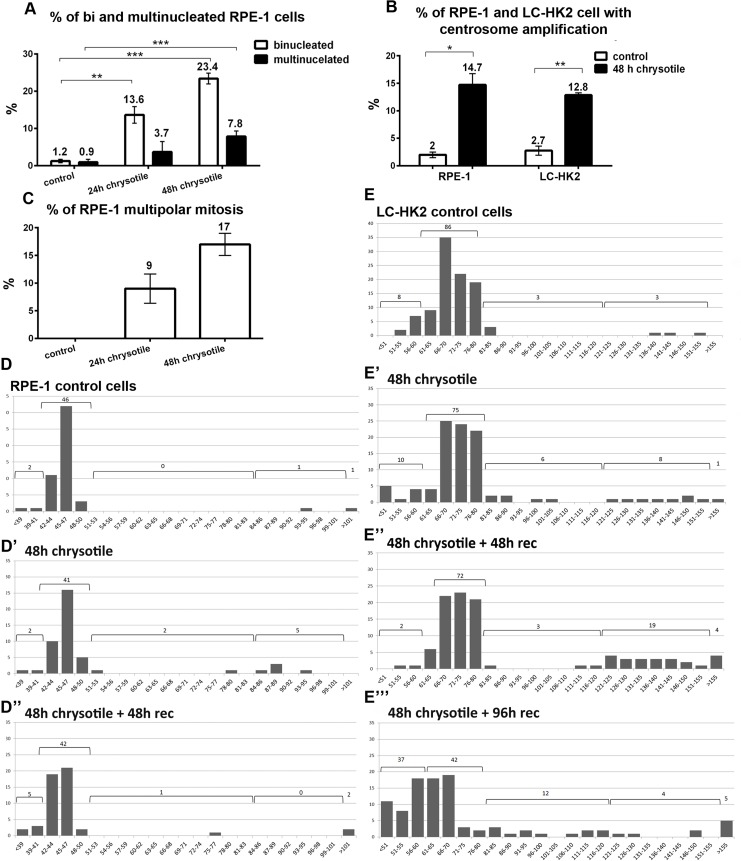
Alterations on cell morphology and ploidy after chrysotile treatment **A**., **B**., **C**. Chrysotile treatment increased the frequencies of bi and multinucleated RPE-1 cells (**A)**, the frequencies of centrosome amplification in RPE-1 and LC-HK2 cells (**B).** and also the frequencies of multipolar mitosis in RPE-1 cells (**C), D.** and **E.** Number of chromosomes in RPE-1 and LC-HK2 cells in metaphase spreads (histograms show number of chromosomes X number of metaphases). **D.** After 48 h of chrysotile treatment, the number of RPE-1 tetraploid cells increased. After 48 h of chrysotile treatment and 48 h of recovery, the numbers of hyperdiploid and tetraploid cells remained similar to control. *n* = 50 metaphases, absolute values are shown. **E.** In control LC-HK2 cells the peak between 61 and 80 chromosomes was considered the diploid state. After 48 h of chrysotile treatment and treatment followed by 48 h of recovery the number of tetraploid metaphases increased. After 96 h of recovery, the diploid peak was reduced and cells with different numbers of chromosomes were observed. *n* = 100 metaphases. * *P* < 0.05, **P < 0.01, and ***P < 0.001.

The main effects of chrysotile treatment on LC-HK2 cancer cells were multinucleation, centrosome amplification and multipolar mitosis, as described by our group [8]. Here we test potential effects of chrysotile on epithelial cells from normal tissue (RPE-1 cells), to compare the behavior of normal and cancer cells after fiber treatment. After 24 h of chrysotile treatment multinucleation (bi+multinucleation) was 8.2-fold higher than control cells and reached 15-fold at 48 h (Figure 1A). At this time point cells with more than two centrosomes increased 7.4-fold over control (Figure 1B), and multipolar mitosis - not observed in control cells- represented 17% of mitotic cells (Figure 1C).

### Chrysotile treatment induces tetraploidy in normal and cancer cells

The number of chromosomes in RPE-1 and LC-HK2 cells was evaluated in metaphase spreads.

92% of mitotic RPE-1 cells had between 42 and 50 chromosomes, considered the diploid interval. The remaining 8% was distributed between less than 42 chromosomes (hypodiploid, 4%), 84 to 101 chromosomes (tetraploid, 2%) and more than 101 chromosomes (hypertetraploid, 2%). After 48 h of chrysotile treatment, the percentages of tetraploid cells increased to 10% and the hyperdiploid to 4%. After treatment followed by 48 h of recovery, the tetraploid and hyperdiploid population decreased, returning to control levels (Figure 1D). After 96 h of recovery, fewer mitosis were observed and no significant differences were observed between control and treated cells (data not shown).

In LC-HK2 cells, tetraploid population was also detected after 48 h of chrysotile treatment and after treatment followed by 48 h of recovery. After 96 h of recovery, the tetraploid population decreased, but at this time point the diploid population was 37%, indicating that fiber treatment led to a dramatically change in chromosome number of proliferating LC-HK2 cells (Figure 1E).

Together, these data showed that chrysotile treatment induced tetraploidy in normal and cancer cells, and that tetraploidy might be a route to aneuploidy mainly in cancer cells.

### Centrosome amplification after chrysotile treatment: a consequence of cytokinesis defects

**Figure 2:**
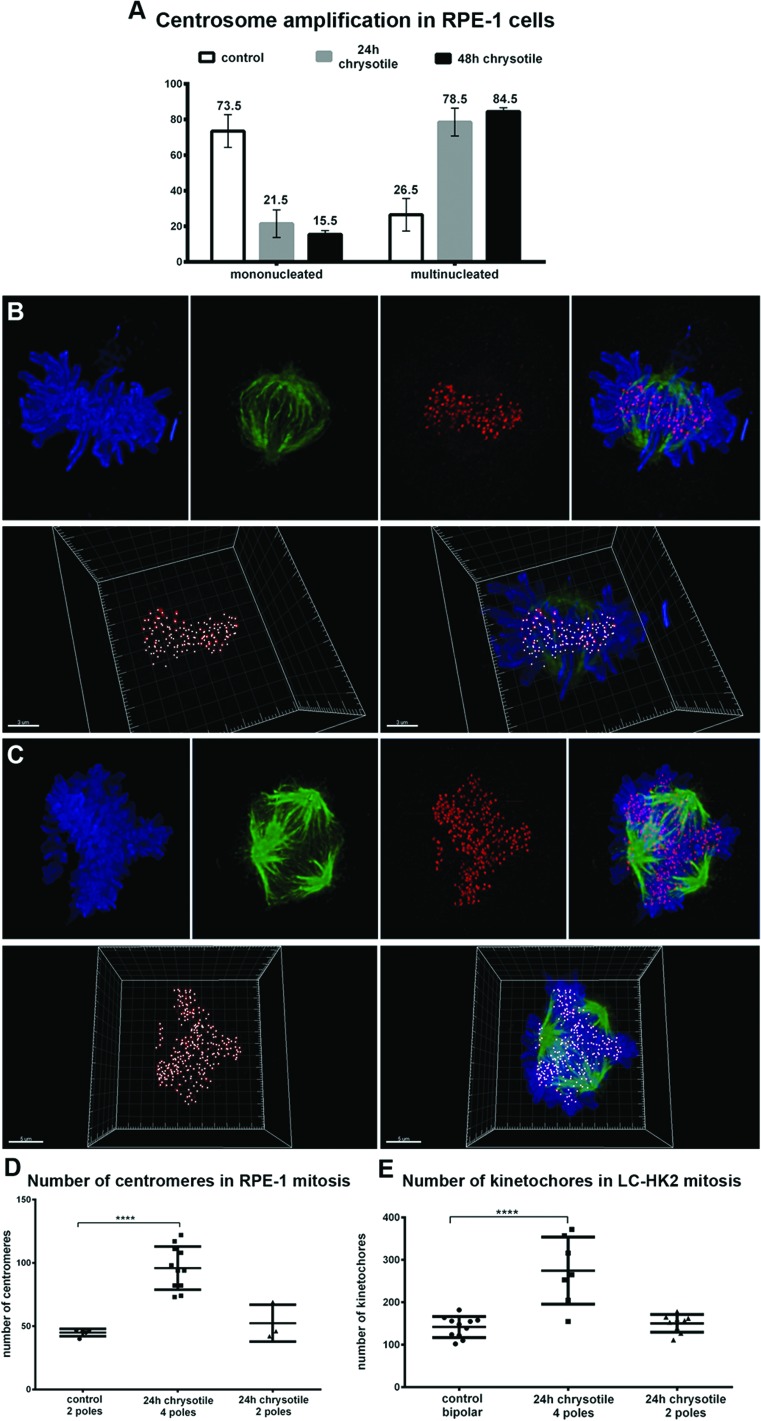
Centrosome amplification in RPE-1 and LC-HK2 cells treated with chrysotile **A.** Considering the number of RPE-1 cells with centrosome amplification as 100%, it was analyzed the distribution of centrosome amplification in mononucleated and multinucleated cells. **B.** and **C.** RPE-1 and LC-HK2 cells were submitted to IF to kinetochores/centromeres (red) and spindle poles detection (αtubulin, in green). Confocal images of metaphases were 3D-reconstructed to evaluate the number of kinetochores/centromeres in bipolar/multipolar mitosis. **B.** Bipolar mitosis of control LC-HK2 cell with 119 kinetochores. **C.** Multipolar LC-HK2 mitosis with 4 spindle poles and more than 300 kinetochores. **D.** and **E.** Multipolar mitoses showed increased number of kinetochores, with average similar to expected for tetraploid cells. *n* = 11 control bipolar mitosis, 7 chrysotile-treated multipolar mitosis and 9 chrysotile-treated bipolar mitoses (D), *n* = 5 control bipolar mitosis, 11 chrysotile-treated multipolar mitosis, 5 chrysotile-treated bipolar mitosis (E). ****P < 0.0001.

After chrysotile treatment, 84.5% of RPE-1 cells with centrosome amplification were multinucleated (Figure 2A). Labeling daughter and mother centrioles evidenced that after chrysotile treatment cells with centrosome amplification generally presented two mother and two daughter centrioles (Figure S1). Similar results were obtained in LC-HK2 cells (data not shown).

To test if centrosome amplification occurred simultaneously with the increase of chromosome number, the number of chromosomes in bipolar and multipolar mitosis was evaluated. Bipolar mitotic control RPE-1 cells showed 40 to 47 centromeres, while multipolar mitotic chrysotile-treated cells showed 73 to 122 centromeres (average of 96) (Figure 2B, 2C and 2E). Similar results were observed in LC-HK2 cells: in control bipolar mitosis it was observed 102 to 182 kinetochores, with an average of 142, and in multipolar mitotic cells after chrysotile treatment 155 to 372 kinetochores were detected, with average of 265 (Figure 2E).

These results showed that centrosome amplification after chrysotile treatment occurred simultaneously to the increase in the number of chromosomes, supporting the idea that centrosome amplification was a result of cell division failure.

### Time-lapse imaging reveals cytokinesis defects and fates of multinucleated cells

LC-HK2 cells expressing H2B-GFP to label chromatin and RPE-1 cells visualized by phase contrast microscopy were examined during mitosis. In control cultures mononucleated cells entered bipolar mitosis that continued into cytokinesis until cells remained interconnected by an intercellular bridge with a phase-dense midbody at the center (Figure S2).

**Figure 3:**
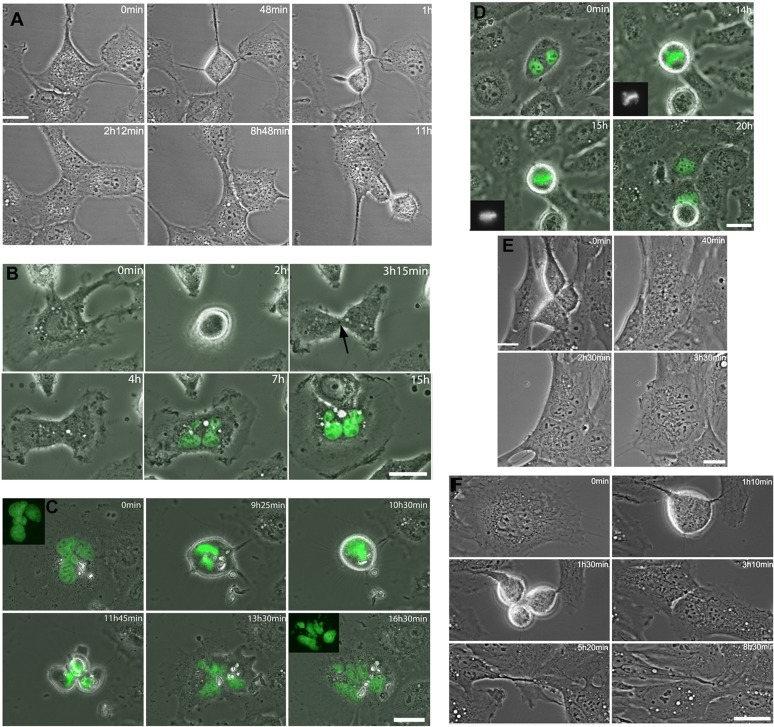
Cytokinesis regression in RPE-1 and LC-HK2 cells Time-lapse imaging of cells treated with chrysotile for 12 to 24 h. **A.** RPE-1 cell entered bipolar mitosis and generated 2 daughter cells. In the intercellular bridge it was observed the midbody and chrysotile fibers. After 7 h in cytokinesis, the cells approached and fused, generating one binucleated cell. **B.** LC-HK2 mononucleated cell entered mitosis and divided into 2 cells. The midbody was observed (arrow), but after 3 h the cells fused into one multinucleated daughter cell. **C.** LC-HK2 multinucleated cell entered multipolar mitosis, divided into 4 daughter cells that fused before the beginning of cytokinesis, generating one multinucleated cell. **D.** LC-HK2 binucleated cell entered multipolar mitosis, after 1 h in metaphase showed its chromosomes aligned and divided into 2 daughter cells. **E.** Multipolar telophase of RPE-1 cell generated 3 cells, but 2 of them fused and during cytokinesis only 2 daughter cells were observed. In the intercellular bridge it was observed chrysotile fibers, and after 1h30min the cytokinesis failed and one multinucleated cell was formed. **F.** Multinucleated RPE-1 cell entered multipolar mitosis, divided into 3 cells that fused generating 2 daughter cells. It was observed fiber in the intercellular bridge.

After 12 h of chrysotile treatment, fibers were often observed in the intercellular bridge (Figures 3A, 3B, S3 and S4). Around 20% of RPE-1 and LC-HK2 cells in mitosis regressed to form a single cell with more than one nucleus after 4 to 16 h in cytokinesis (Figure 3A, 3B, and Figure S3). In some cases the intercellular bridge seemed to be resolved, but the daughter cells approached or emitted new protrusions and then fused (Figure S3). Some cells with fibers in the intercellular bridge could finish cytokinesis, delivering the fiber to one of the cells after severing of the bridge in one point (Figure S4A). Other possibility was the fiber release in the extracellular medium after bridge severing (Figure S4B).

After 24 h of chrysotile treatment, multinucleated cells were observed in LC-HK2 and RPE-1 cultures. RPE-1 multinucleated cells could not start mitosis at similar rates than mononucleated cells, while LC-HK2 multinucleated cells entered mitosis as mononucleated cells did. The multinucleated cells that entered mitosis showed similar fates in both cell lines. These cells progressed through cell cycle and in some cases formed bipolar spindles after organizing their chromosomes in the metaphase plate (Figure 3D). However, in most cases when a multinucleated cell progressed through cell cycle they generated multipolar mitoses. These cells could be divided into 2 to 4 daughter cells, often followed by cell fusion, generating bi or multinucleated cells (Figure 3C, 3E and 3F). Other possibility was the cell division in three cells followed by cell fusion generating two daughter cells, which could finish cytokinesis properly (Figure 3F) or undergo cytokinesis regression.

According to these data, multipolar mitoses after chrysotile treatment were often generated by multinucleated cells that could progress through cell cycle. These multipolar mitoses often resulted in cell fusions that generated other multinucleated cells.

### Disruption of cytokinetic proteins after chrysotile treatment

To investigate alterations during cytokinesis following chrysotile treatment, cells were treated with fibers for 24 h or 48 h, fixed and imaged to determine the location of proteins involved at different cytokinetic stages.

**Figure 4:**
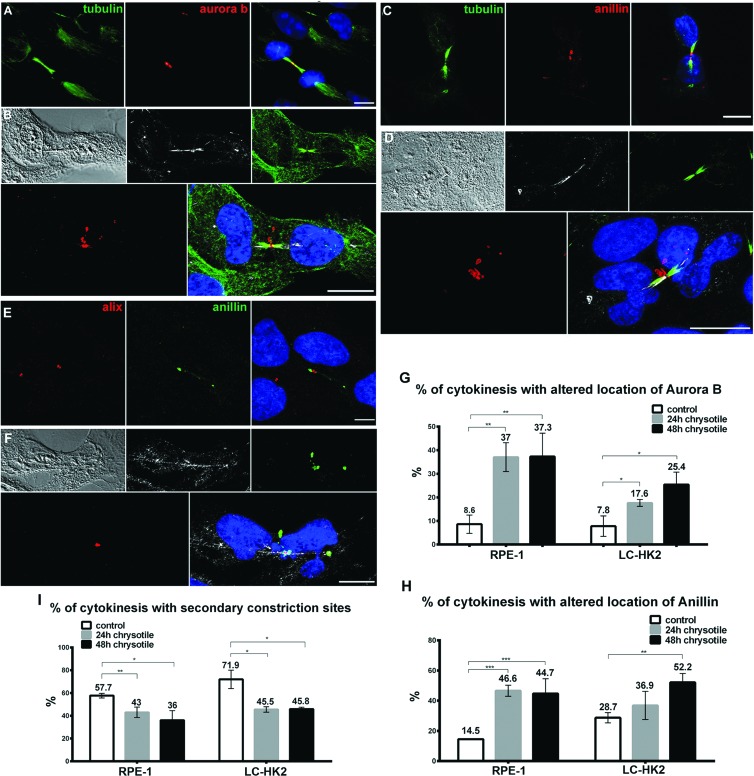
Mislocalization of proteins during cytokinesis RPE-1 and LC-HK2 cells were treated with chrysotile for 24 h and submitted to IF with anti-aurora B, anti-Anillin, anti-Alix, anti-αtubulin or anti-acetylated tubulin antibodies. Cells' nuclei were stained with DAPI (blue), chrysotile fibers were detected by its autofluorescence (white) and DIC images were taken to improve the visualization of fibers and cell shape. **A.** In control RPE-1 cells, Aurora B was restricted to the flanking zones during cytokinesis. **B.** Altered location of Aurora B during cytokinesis of RPE-1 cell with fiber in the intercellular bridge. **C.** Most of control RPE-1 cells showed Anillin in the constriction sites, near the stem body. **D.** Anillin located in different structures in the intercellular bridge of RPE-1 cell, most of them near the stem body and cell cortex. **E.** Alix was detected only in late stages of cytokinesis, in 2 parallel structures near the stem body. **F.** After 24 h of chrysotile treatment Alix was observed in only one structure in RPE-1 cells. **G.** and **H.** Chrysotile treatment increased the number of intercellular bridges with altered location of Aurora B (G) and Anillin (H) in RPE-1 and LC-HK2 cells. **I**. After chrysotile treatment, the percentages of intercellular bridges with constrictions sites decreased in both cell lineages. *n* = 100 cytokinesis in 3 different experiments. *P < 0.05, **P < 0.01, and ***P < 0.001.

Aurora B is a kinase located at midzone during telophase and at the flanking regions of the midbody during cytokinesis [27]. When Aurora B is inactivated cytokinesis can progress and abscission occurs. On the other hand, activation of Aurora B inhibits late stages of cytokinesis [28, 29]. In control RPE-1 and LC-HK2 cells Aurora B was located properly (∼90% of cytokinesis, Figure 4A and 4G). After 24 h and 48 h of chrysotile treatment, Aurora B was not restricted to midbody flanking regions. In 20-40% of cytokineses it appeared as rings and puncta in the regions between the midbody and the plasma membrane, with a dramatic concentration at the midbody ring/stem body (Figure 4B and 4G).

Anillin (actin binding protein) localizes at the midzone near the plasma membrane during telophase, and then forms plasma membrane tubular protrusions at the intercellular bridge during its establishment. At late stages of cytokinesis Anillin is located at the stem body [27, 30], and then is directed to the secondary constriction sites [31]. The roles of Anillin are not fully understood; however, it seems to be crucial for furrow ingression establishment [30] and abscission sites determination [31, 32]. In control RPE-1 and LC-HK2 cells Anillin was observed in the intercellular bridge forming one ring in the stem body or could be located in the stem body and secondary constriction sites. In 14.5% and 28.7% of the cytokinesis of RPE-1 and LC-HK2 cells, respectively, location of Anillin was not restricted to constriction sites or stem body, and others structures in the intercellular bridge were detected (Figure 4C and 4H). After 24 h of chrysotile treatment Anillin was observed in larger structures similar to rings near the stem body or near the plasma membrane (Figure 4D). These structures occurred in almost 50% of RPE-1 cytokineses after 24 h and 48 h of chrysotile treatment, and in LC-HK2 cells they were observed 37% of cytokinesis after 24 h of treatment and reached 52% 48 h of treatment followed by 24 h of recovery (Figure 4H).

Other proteins were investigated only in RPE-1 cells. Septin 9 is member of the Septin protein family that is involved in abscission. It is located similarly to Anillin during cytokinesis, and is also involved in tubular structures in initial steps of cytokinesis and secondary constriction site determination [31, 32]. It was detected in control cells at the midbody, near the stem body or secondary constriction sites (Figure S5A). After chrysotile treatment for 24 h, Septin 9 was present in the midbody in huge structures from the stem body to the plasma membrane (Figure S5B and C). Alix, a protein required for abscission [33], appears at midbody during intermediate and late stages of cytokinesis and it is directed to the abscission site when it is determined. In RPE-1 cells Alix was observed in two parallel structures in the midbody (Figure 4E). After 24 h or 48 h of chrysotile treatment Alix was observed in just one structure in center of the midbody (Figure 4F).

In contrast to the mislocalization of Aurora B, Anillin, Septin 9 and Alix, other proteins were normally localized in the intercellular bridge after chrysotile treatment. Cenp E (centromere protein E) is located at the midzone during telophase and then is directed to flanking zones at the midbody. RacGAP1 (a GTPase activating protein involved in RhoA regulation) is directed to the stem body during cytokinesis. MKLP1 is a marker of the midbody, in a ring in the stem body observed during cytokinesis and in post-mitotic midbodies. These three proteins - Cenp E, RacGAP1 and MKLP1 - were properly located in the intercellular bridge in chrysotile-treated cells (data not shown). This indicated that chrysotile treatment did not alter all stages of cytokinesis and location of all cytokinetic proteins, but mainly the ones related to intermediate and final steps of cytokinesis.

The presence of secondary constriction sites - a late step of constriction of the intercellular bridge during cytokinesis - was seen after IF staining for acetylated tubulin. In control RPE-1 and LC-HK2 cells 58% and 72% of intercellular bridges showed secondary constriction sites, respectively. After 24 h of chrysotile treatment, the presence of secondary constriction sites decreased to 43% of the cytokinesis in RPE-1 cell and 45% in LC-HK2 cells. During longer treatment periods and after treatments followed by recovery periods the percentages of intercellular bridges with secondary constriction sites remained reduced relative to controls (Figure 4I).

### Chrysotile altered expression of genes related to cell cycle and cytokinesis

The expression of genes related to cell cycle and cytokinesis was evaluated, and expression was always showed relative to that in control cells.

**Figure 5:**
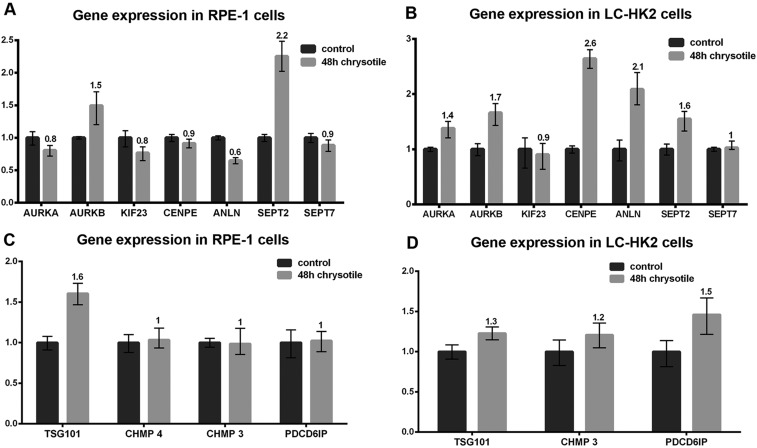
Expression of genes related to cell cycle and cytokinesis The expression of genes related to cell cycle and cytokinesis was analyzed by quantitative PCR. The expression in treated cells were evaluated in relation to control cells. **A.** and **B.** Expression of genes related to cell cycle and early and intermediate stages of cytokinesis. RPE-1 cells treated with chrysotile for 48 h (A) showed increased expression of the genes that encode Aurora B and Septin 2, and decreased expression of Anillin. LC-HK2 cells treated with chrysotile (B) showed increased expression of Aurora A, Aurora B, Cenp E, Anillin and Septin 2 genes; **C.** and **D.** Expression of genes related to final stages of cytokinesis and abscission. RPE-1 chrysotile-treated cells showed increased expression of TSG101 gene LC-HK2 cells treated with chrysotile showed increased expression of the gene that encodes Alix.

In both cells lines chrysotile treatment for 48 h resulted in increased expression of AURKB and SEPT2 genes, which encode Aurora B and Septin 2 proteins. Increased expression of ANLN (Anillin gene) was detected in LC-HK2 cells and decreased expression of this gene was observed in RPE-1 cells. In LC-HK2 cells, CENPE and AURKA showed increased expression, while no difference was observed in CENPE and AURKA expression in RPE-1 cells. No differences in expression of SEPT7 (Septin 7 gene) and KIF23 (which encodes MKLP1 protein) were observed after chrysotile treatment in either cell line. Among genes related to abscission, RPE-1 cells showed increased expression of TSG101 (a member of ESCRT-I complex, required for membrane fusion and fission) [33], while LC-HK2 cells showed increased expression of PDCD6IP - the gene that encodes Alix. No differences in expression of CHMP4 and CHMP3 (ESCRT complex proteins, related to membrane budding and fission) [33] were detected in both cells lines (Figure 5).

These data showed that chrysotile treatment affected the expression of some genes related to cytokinesis and abscission in RPE-1 and LC-HK2 cells. The number of altered genes was higher in LC-HK2 cells - a cancer cell line. The differences in genes expression did not exceeded 2.5 fold, indicating that the treatment did not lead to striking differences in expression. However, the differences in genes related to cytokinesis indicated that a cellular response to chrysotile internalization might include changes in gene expression, and that these differences may interfere with cell behavior.

### Chrysotile treatment alters proliferation of normal cells

**Figure 6:**
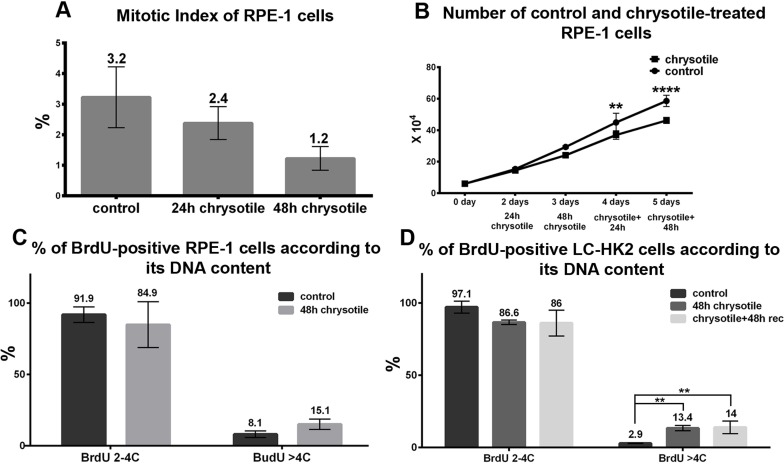
Cell cycle and proliferation in RPE-1 and LC-HK2 cells after chrysotile treatment **A.** RPE-1 mitotic index after 24 and 48 h of chrysotile treatment. **B.** Growth curve of RPE-1 cells. The number of cells in culture decreased after 48 h of treatment and 48 h of recovery when compared to control. **C.** and **D.** The percentages of cells in S phase was evaluated by BrdU incorporation. The treatment did not led to differences in the number of cells in S phase in both cell lineages. However, when the BrdU-positive cells were analyzed according to its DNA content, it was observed increased numbers of hypertetraploid cells in S phase in LC-HK2 cells. **P < 0.01, and ****P < 0.0001.

Despite morphological alterations caused by chrysotile in LC-HK2 cells, they do not show a reduction of mitotic index or number of cells in culture [25]. In RPE-1 cells the mitotic index decreased from 3.2 to 1.2% after 48 h of treatment (Figure 6A), and the number of cells in culture decreased 0.7-fold after 48 h of treatment followed by 48 h of recovery (Figure 6B).

The proliferation of LC-HK2 and RPE-1 cells was analyzed by BrdU incorporation. The percentages of RPE-1 and LC-HK2 cells in S phase did not show significant differences after chrysotile treatment. However, when the percentages of cells in S phase were analyzed by their DNA content, the percentages of cells in S phase with DNA content higher than 4C increased mainly in LC-HK2 cells: in RPE-1 cells treated with chrysotile for 48 h, we saw a 1.9-fold increase of cells in S phase with more than 4C DNA content over control, while in LC-HK2 cells this increase was about 4.6-fold over control cells (Figure 6C and 6D). This data showed that RPE-1 and LC-HK2 cells, diploid or not, were able to enter S phase, but RPE-1 showed limited capability to enter M phase after chrysotile treatment.

## DISCUSSION

Chrysotile fibers were reported to lead to aneuploidy *in vitro* and *in vivo*, but how aneuploid cells arise was not clear. There was some evidence regarding cytokinesis defects, centrosome amplification and multipolar mitosis, but no connection between these abnormalities and generation of aneuploid cells was made. In the present study we investigated the causes of centrosome amplification and our data demonstrate that cytokinesis failure might be the major responsible for this amplification. Corroborating this idea, the first alteration after fiber treatment observed by time-lapse was cytokinesis regression. Investigating the causes of this regression, we observed that specific proteins required for cytokinesis were mislocated in the intercellular bridge after fiber exposure, and that some genes related to cytokinesis were differentially expressed. Also, we observed that tetraploid cells generated in normal and cancer cell lines could progress through cell cycle and originate multipolar mitoses, which can form aneuploid cells. Aneuploid cancer cells, but not aneuploid normal cells, were observed in mitosis, indicating that in normal-derived cells tetraploidy does not limit proliferation, while aneuploidy does. This data is summarized in Table 1.

**Table 1:** Alterations in RPE-1 and LC-HK2 cells after chrysotile treatment

	ALTERATION	RPE-1 CELLS	LC-HK2 CELLS
Cell morphology	Bi and multinucleation	increased	increased
	Centrosome amplification and multipolar mitosis	increased	increased
Ploidy	Tetraploidy in metaphase spreads	increased	increased
	Aneuploidy in metaphase spreads	no change	increased
	Correlation between centrosome amplification and increased number of chromosomes	present	present
Proliferation	Growth curve after treatment and recovery	decreased	no change
	Mitotic index	decreased	no change
	Cells in S phase	no change	no change
Cytokinesis	Presence of secondary constriction sites	decreased	decreased
	Altered location of cytokinetic proteins	increased	increased
	Cytokinesis regression	increased	increased

In our experiments, chrysotile fibers were internalized by RPE-1 and LC-HK2 cells in less than 24 h. The first alteration in mitosis was observed during cytokinesis, as reported previously [8, 26]. New fates of fibers located in the intercellular bridge were observed: one of the daughters could incorporate them following abscission or the fibers could be released into the culture medium with part of the intercellular bridge. However, in most cases of cytokinesis with a fiber in the intercellular bridge the fate was regression followed by generation of a multinucleated daughter cell.

Some studies show the potential of asbestos fibers to bind and retain proteins when incubated with cell lysates or serum [9, 17], and this process can also increase fiber uptake by cells [34]. However, the images in the present study did not show proteins required for cytokinesis co-localized or retained by fibers. This indicates that either fibers did not adsorb these proteins or that fibers were surrounded by membrane inside the cells, and did not interact with the cytoplasm and its proteins. Chrysotile fibers are also capable to adsorb phospholipids and membranes [35, 36], which could help to maintain fibers surrounded by membranes and not in contact with the proteins in the cytoplasm.

The presence of fibers did not affect the mechanisms responsible for recruitment of proteins to intercellular bridge. However, the localization of some proteins - Anillin, Septin 9, Aurora B and Alix - was altered in chrysotile treated cells. These proteins are involved mainly in intermediate to final steps of cytokinesis. Anillin is important for Septin recruitment into filaments near to the plasma membrane. After intracellular bridge maturation these protein migrate to the stem body or to secondary constriction sites, where they are crucial for determination of the abscission site, where the ESCRT III complex will act [31]. Aurora B also coordinates cytokinesis - its activation delays cytokinesis and abscission -, and it has been related to abscission by phospholyration of ESCRT elements [37]. In chrysotile-treated cells with mislocalization of Septin, Anillin and Aurora B, bridges showed thickness and length similar to mature bridges, but Anillin and Septin did not migrate to constriction sites. The number of bridges with secondary constriction sites decreased, and Alix, a protein related to abscission, did not migrate to abscission sites, supporting the idea that Anillin and Septin mislocalization results in abscission failure. The presence of chrysotile fibers may not allow bridge maturation, maintaining a molecular signal which results in no secondary constriction sites. This signal could be related to Anillin and Septin since they are required for abscission site determination, and also to Aurora B, which is involved in the No Cut pathway [28, 38]. Together, these results suggested a cellular response, which involves Aurora B, Anillin and Septin in delaying abscission, what should be further investigated.

The expression of some genes related to cytokinesis was altered after chrysotile treatment in RPE-1 and LC-HK2 cells. Although chrysotile was reported to interact with RNA and proteins related to transcription and translation, being able to alter these processes [19], the differences found in gene expression in the present study might be a consequence of cytokinesis defects. The presence of chrysotile fibers in the intercellular bridge and the lack of constriction sites specification may induce cellular responses that include changes in gene expression. The increased expression of AURKB and SEPT2 in RPE-1 and LC-HK2 cells after chrysotile treatment may be part of a mechanism that prevents cytokinesis progression.

Interestingly, overexpression of Septins and Aurora B were described in many solid tumors, such as lung, brain and breast [39, 40]. Aurora B is involved in different mitotic processes, and is now a target of new cancer therapies [40, 41]. Overexpression of Septin 9 was widely detected in tumors [39] and SEPT9 is now considered a biomarker for early detection of colorectal cancer [42, 43]. Septin 2 is involved in cytokinesis and chromosome alignment, and can also be involved with aneuploidy promotion [44]. In this context, the mislocation of Septin 9 and Aurora B and the overexpression of SEPT2 and AURKB gene in chrysotile-treated cells may represent another tumoral cell feature acquired by the presence of fibers. Moreover, our data show that Septins and Aurora B mislocation and/or overexpression might be related to cytokinesis failure and generation of aneuploidy, revealing a possible role of these proteins in cancer development.

Importantly, all the alterations observed during cytokinesis culminated not only in cytokinesis failure, but also in regression and tetraploid cell formation with centrosome amplification. Tetraploid cells from both studied cell lines progressed through cell cycle and reached mitosis, generating multipolar mitoses. Different abnormalities during these mitoses were observed, leading to aneuploidy. It was during mitosis that a difference between normal and cancer-derived cells was observed: aneuploid cells in mitosis were only observed in cancer cells.

Control cancer cells already showed aneuploid DNA content, so differences in these aneuploid karyotypes might not be so deleterious when compared to normal cells. However, these differences also showed that cancer cells can be adapted to several differences in their karyotype. In normal RPE-1 cells, aneuploidy interfered with proliferation, and aneuploid cells were not observed in metaphase spreads. Normal cells might have mechanisms to detect karyotypes different from diploid and tetraploid and prevent these cells proliferation. Alternatively, aneuploid karyotypes could generate an imbalance of RNA and proteins that decreases cell viability and proliferation. In this second case, RPE-1 aneuploid cells after chrysotile treatment may proliferate at low rates, reducing the chance to be detected in our *in vitro* experiments.

Together, these data showed that chrysotile fibers can induce cancer-associated characteristics in normal epithelial cells, and can increase the frequency of these properties in cancer cells. We found that aneuploidy generated after chrysotile treatment was a result of cytokinesis regression and tetraploidization, probably due to a mechanism that involved mislocalization of Aurora B, Anillin and Septins, and lack of constriction and abscission site specification. The tetraploid as a state between diploid and aneuploid karyotypes has been described, and represent a route to cancer development [5, 6, 45, 46]. In this context, our findings show that cancer development after chrysotile exposure may be promoted after cytokinesis failure. Chrysotile fibers have been also related to production of reactive oxygen species [47] and DNA strand breaks [21, 23, 48]. These alterations, together to the generation of aneuploid cells, create a very favorable context to cancer development and show a possible way of how a mineral fiber can interfere with cell behavior and promote cancer-associated abnormalities.

## MATERIALS AND METHODS

### Cell culture

The LC-HK2 cells (initially called HK2, a cell line established from human non-small cell lung carcinoma) [49] and hTERT RPE-1 (RPE-1) cells (derived from normal human retina epithelium - ATCC CRL-4000) were cultured in Dulbecco's Modified Eagle's Minimum Essential Medium (Sigma, St Louis, MO, USA), supplemented with 10% fetal bovine serum, in a humidified atmosphere with 5% CO2 at 37°C.

### Chrysotile treatment

Chrysotile 5R (Quebec Standard) obtained from SAMA Mineração de Amianto Ltda (Minaçu, GO, Brazil) were provided by Dr. Flavia M. Cassiola. The fibers were prepared as describe elsewhere [50], and were added to culture medium. Treatments were performed 35 mm diameter dishes (2.10^5^ cells/dish). After 24 h in culture, cells were treated with 2 mL of fresh medium with 125 μg/ml of chrysotile fibers. After 24 h or 48 h the medium was changed and additional periods of 48 h, 3 or 4 days in fiber-free medium were conducted (called recovery periods).

### Metaphase spreads

LC-HK2 and RPE-1 cells were treated with 0.01% Vincristine for 1 h, enzymatically removed from the flasks and treated with hypotonic solution (4 mL of 0.072M KCl and 6 m L of 1% sodium citrate) for 20 min. Cells were fixed in methanol:acetic acid (3:1) and placed into histological slides. After 3 days cells were stained with Wright (Merck, Germany) diluted in phosphate buffer (3:1). Cells were observed under AxioVert microscope (Carl Zeiss, Germany) with 100X objective. 100 metaphases of LC-HK2 cells and 50 metaphases of RPE-1 cells were analyzed in three different slides from different experiments.

### Flow cytometry

Cell cycle analysis was by flow cytometry, using Guava System (GE Healthcare, UK). Cells were detached with trypsin, fixed with methanol: PBS (3:1) for 1 h at 4°C and incubated with a solution of 200 μl of PBS, 20 μl of 10mg/mL RNAase and 20 μl of PI for 1 h. 5,000 cells were analyzed for each control and treated sample. BrdU incorporation was performed with BrdU FITC Flow Kit (BD Biosciences, USA), according to manufacturer's protocol.

### Immunofluorescence (IF)

Control and treated cells were fixed with 3.7% formaldehyde for 30 min or cold methanol for 15 min, and treated with PBSAT (PBS, 0.1% Triton X-100, 1% BSA) for 30 min. Then the cells were stained for immunofluorescence with primary antibodies for 1 h followed by 45 min with secondary antibodies (details in Table S1). The cells nuclei were stained by DAPI. Cell morphology and presence of chrysotile fibers were imaged by laser scanning confocal microscopy (LSM 710, Carl Zeiss). These preparations were also used to quantify multinucleated, mitotic index and multipolar mitosis: preparations were observed using fluorescence microscopy and 1,000 cells/slide and 100 mitosis in three different slides were counted for each treatment and control. For kinetochore quantification, confocal images were 3-D reconstructed by Imaris software (Bitplane).

### Time-lapse microscopy

LC-HK2 were transfected (Lipofectamine 2000, Invitrogen, USA, performed according manufacturer's protocol) with the H2B-GFP plasmid to allow chromatin observation. After 24 h chrysotile treatment started. Control and treated cells were observed by time-lapse microscopy in a Nikon Biostation microscope (Nikon, Japan). RPE-1 cells were observed in a Zeiss Axioskop 2 microscope, a Zeiss Axiovert 200 microscope with a PerkinElmer UltraView LAS spinning discwith a 40X or 63X objective, with controlled temperature. The control and chrysotile-treated cells were observed for 18 h to 24 h.

### RNA extraction and quantitative PCRs

RNA was extracted using the ChargeSwitch total RNA Cell Kit (Invitrogen) and quantified using a NanoDrop ND1000 Spectrophotometer. The expression profile was determined by real-time RT-PCR analysis (Corbett Research - Rotor Gene 6000 real-time cycler) using an AgPath-ID One-Step RT-PCR kit (Ambion) and SYBR Green (Invitrogen). The qRT-PCR conditions were as follows: 45°C for 10 minutes, 95°C for 15 minutes and 35 cycles [95°C for 15 seconds; Tm°C for 20 seconds; 72°C for 30 seconds], followed by the melt. Primers details are in Table S2. The normalization was calculated using the total RNA [51, 52].

### Statistical analyses

The results were analyzed by Student's *t*-test and *P* < 0.05 was considered significant. In figure legends: *P < 0.05, **P < 0.01, ***P < 0.001 and ****P < 0.0001.

## SUPPLEMENTARY MATERIAL FIGURES AND TABLES


