# Supplementary material for: Multipolar mitosis and aneuploidy after chrysotile treatment: a consequence of abscission failure and cytokinesis regression
Source: Oncotarget. 2016 Jan 15;7(8):8979–92. doi: 10.18632/oncotarget.6924 (PMC4891019; doi:10.18632/oncotarget.6924)
Supplement: Supplementary file 1 [file oncotarget-07-8979-s001.pdf]

Multipolar mitosis and aneuploidy after chrysotile treatment: a consequence of abscission failure and cytokinesis regression

Supplementary Material

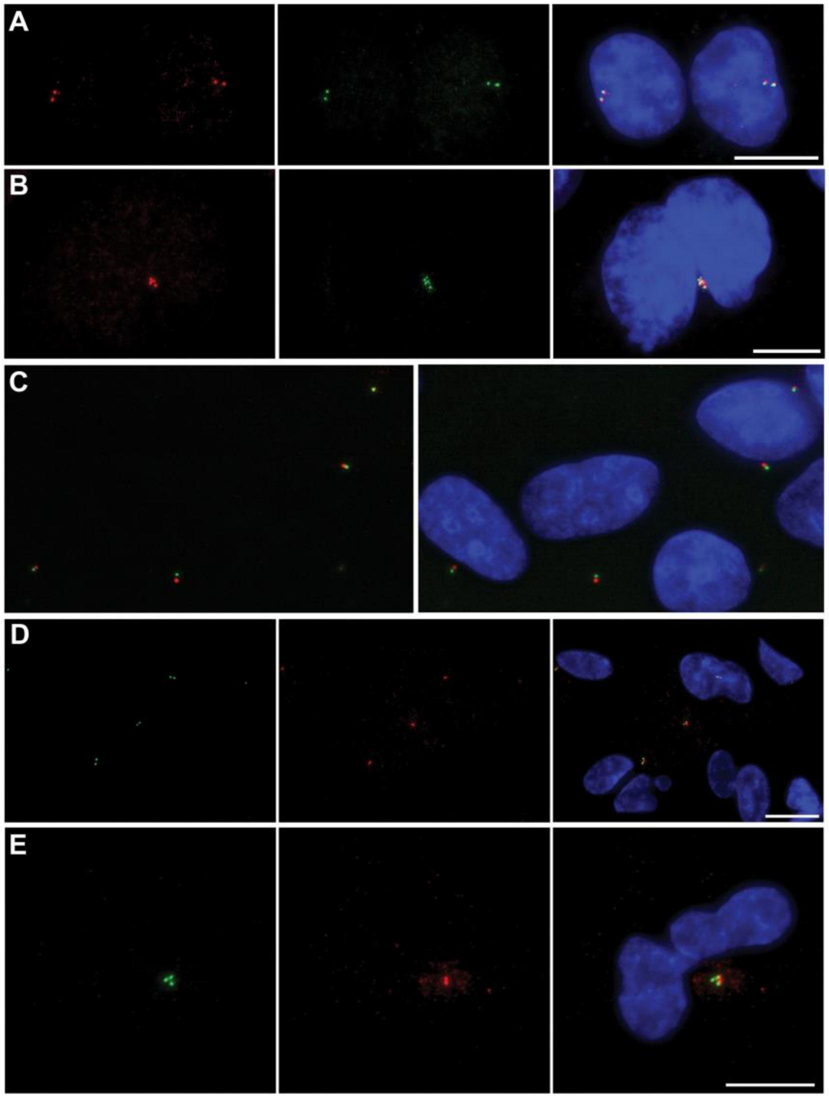

Figure S1. Centrosome morphology in RPE-1 cells. RPE-1 control and chrysotile-treated cells were submitted to IF and centrosomes and centrioles were analyzed. A) Control cells were mononucleated with one centrosome formed by two centrioles (gamma tubulin in red, centrin in green). B) Binucleated cell formed after 24 h of chrysotile treatment with more than 4 centrioles (gamma tubulin in red, centrin in green). C) Control cells with one mother centriole (cenexin, in red) and one daughter centriole (centrobin, in green). D and E) Cells after 24 h of chrysotile treatment. Multinucleated cells with two daughter and two mother centrioles (D) and with three daughter and two mother centrioles (E). Bar=10µm.

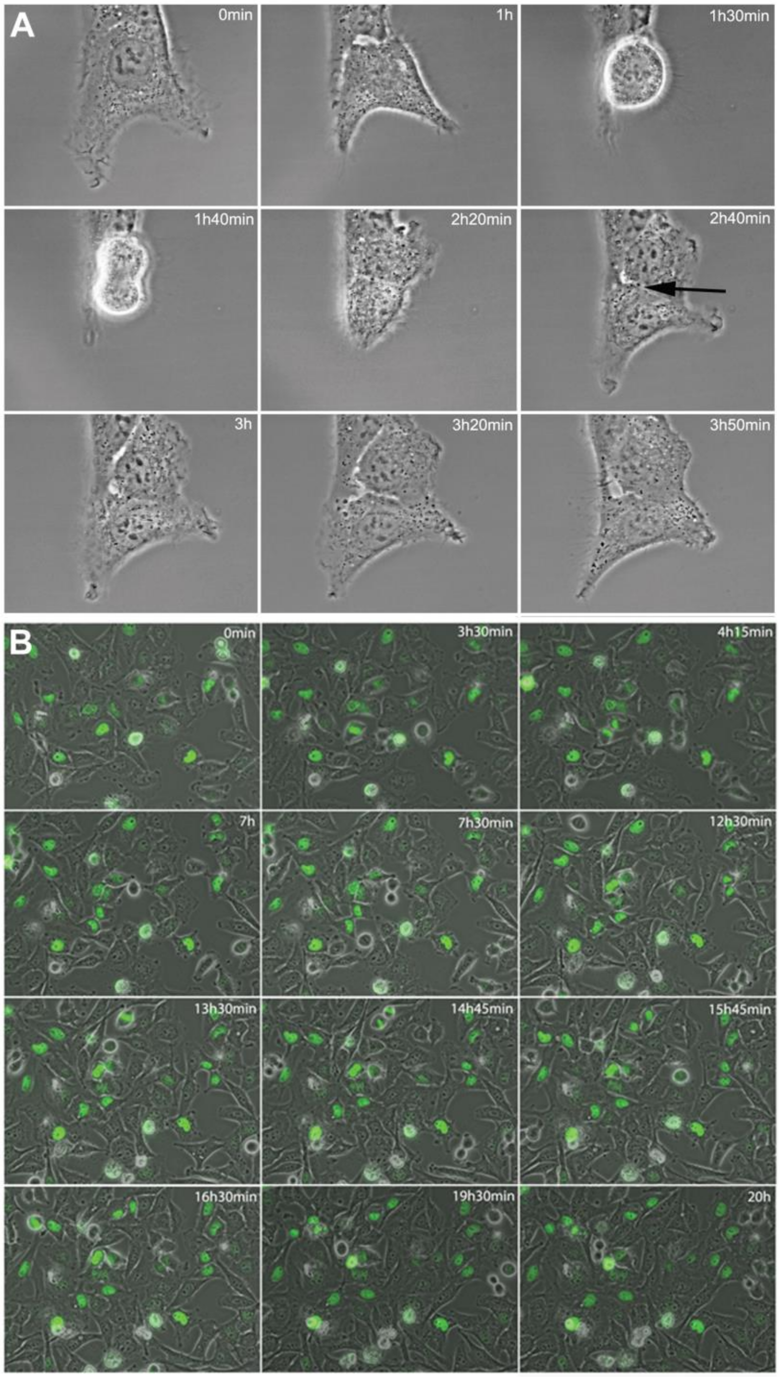

Figure S2. Time-lapse imaging of control RPE-1 and LC-HK2 cells. A) In control RPE-1 cultures mononucleated cell entered bipolar mitosis and generated two daughter cells. The midbody was evident during cytokinesis (arrow). B) LC-HK2 cells transfected with H2B-GFP plasmid were imaged by time-lapse. Cells expressing or not the fused protein entered mitosis in similar rates. Most of mitosis generated two daughter cells.

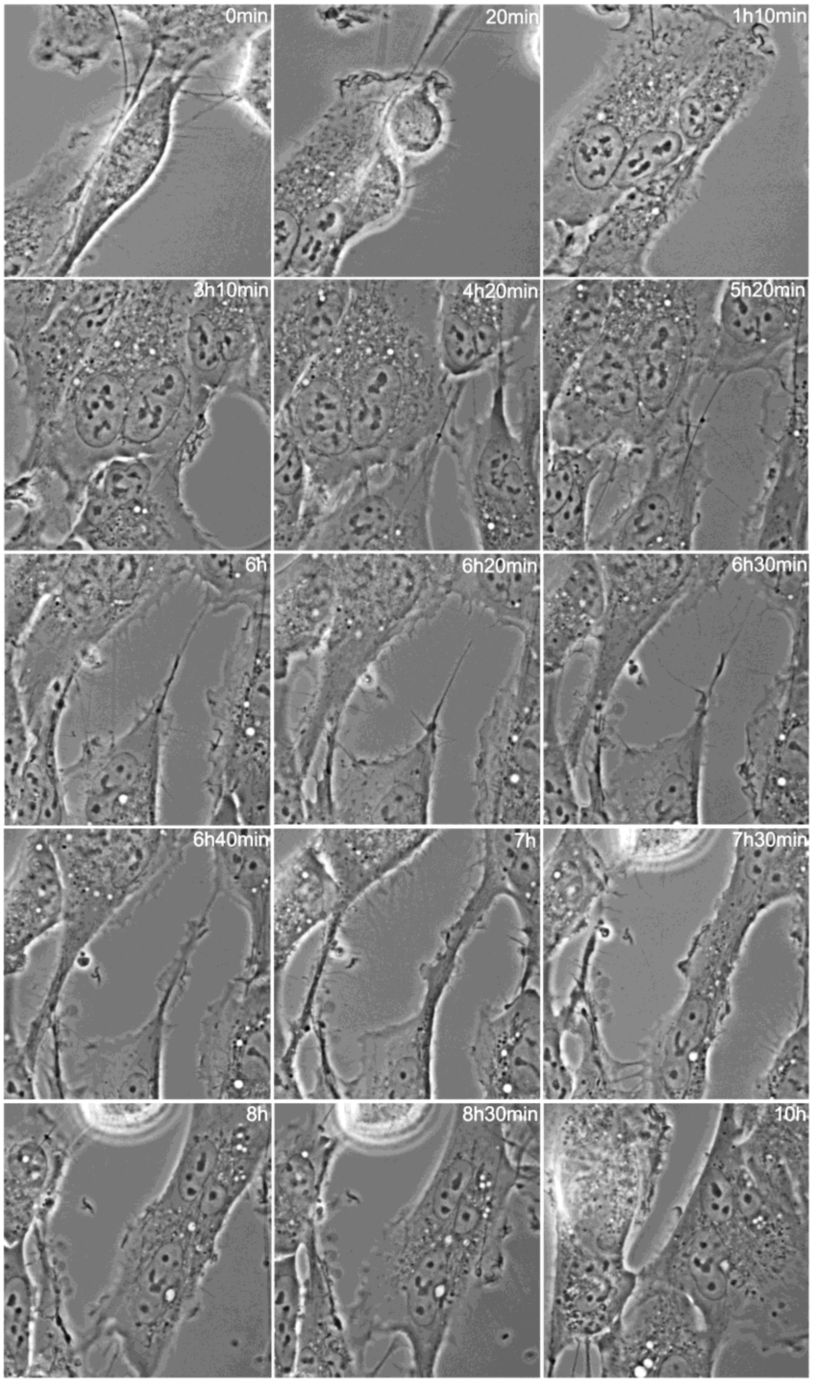

Figure S3. Cytokinesis regression in RPE-1 cells. RPE-1 cell bipolar after mitosis formed intercellular bridge with midbody and chrysotile fiber. After 6 h the midbody was directed to one of the daughter cells, and cytokinesis seemed to be finished. However, cells emitted membrane protrusions and approached, generating only one multinucleated daughter cell.

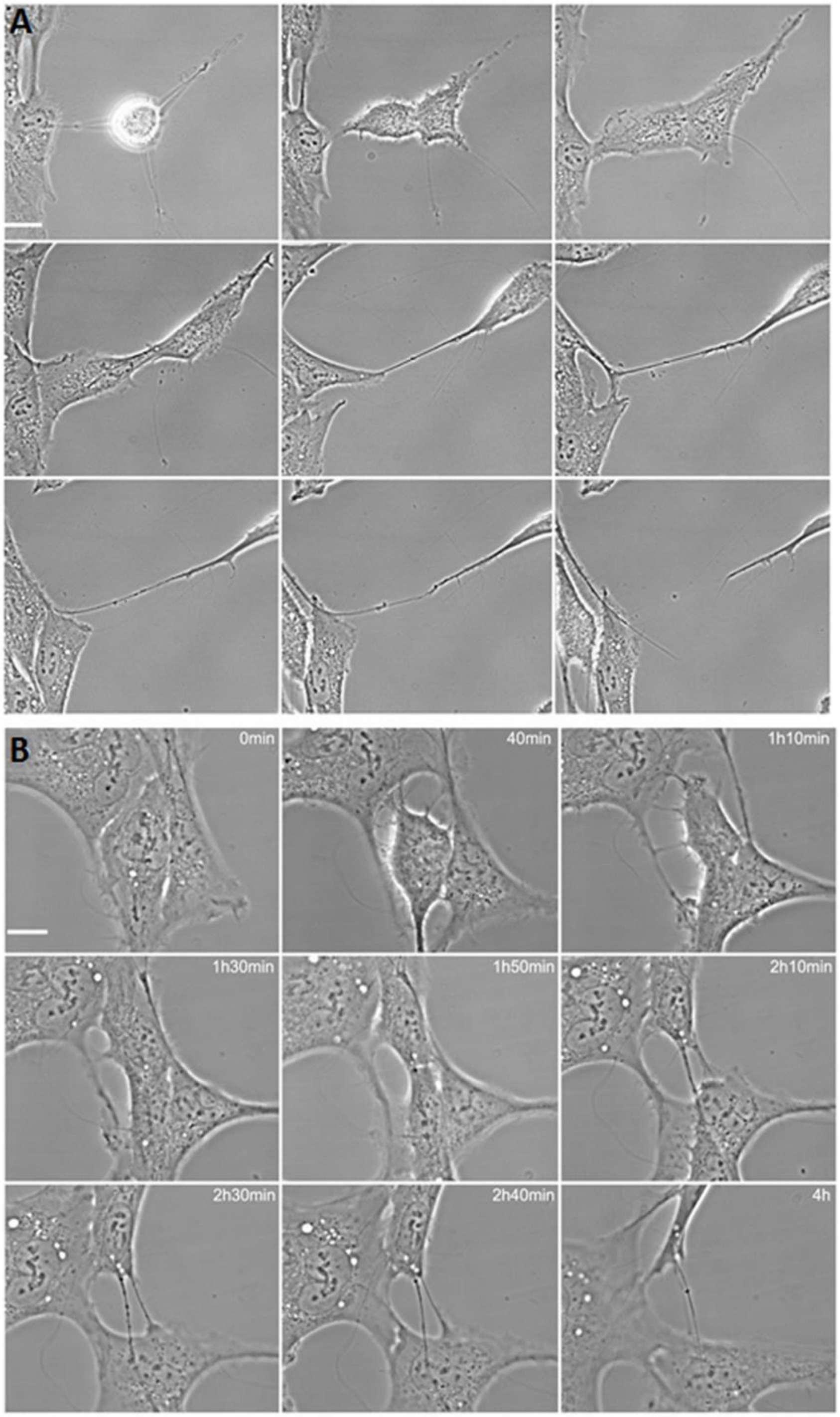

Figure S4. Cytokinesis of RPE-1 cells with chrysotile fibers in the intercellular bridge. RPE-1 cells were treated with chrysotile for 12 h and observed by time-lapse imaging. A) Bipolar mitosis with a fiber in the intercellular bridge. The bridge became very thin and long, when it was disrupted in one point. The fiber was directed to one of the daughter cells. B) Mononucleated cell entered a bipolar mitosis, and formed two daughter cells connected by an intercellular bridge with a chrysotile fiber. The bridge was cleaved in two distant points, and the bridge with the fiber was released in the extracellular medium.

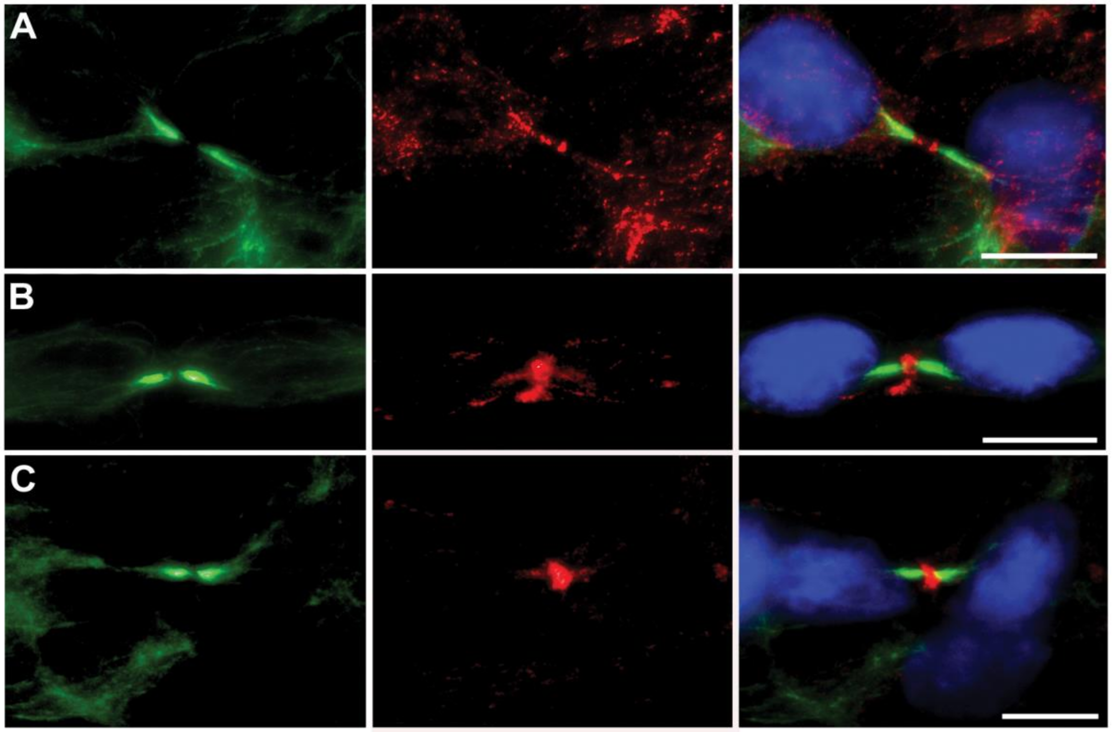

Figure S5. Location of Septin 9 in control and chrysotile-treated RPE-1 cells. RPE-1 cells were submitted to IF with anti-septin9 antibody (red). Nuclei in blue and microtubules in green. A) In control RPE-1 cells Septin9 was located in the stem body or near the secondary constriction sites in the intercellular bridge. B and C) After 24 h of chrysotile treatment Septin 9 was observed near the midbody in the intercellular bridge, but in huge structures directed to the plasma membrane.

Table S1: Antibodies used for IF.

Antibody	Dilution
Anti-alpha tubulin (Sigma)	1:200
Anti-aurora B (Abcam, ab2254)	1:50
Anti-anillin (Santa Cruz, sc-67327	1:200
Anti-alix (Santa Cruz, sc-53539)	1:150
Anti-cenpE (Abcam, ab5093)	1:200
Anti-centrin (Millipore 04-1624)	1:10,000
Anti-centrobin (Abcam, ab70448)	1:200
Anti-cenexin (Protein Tech, 12058-1-AP)	1:200
Anti-Hec1 (Abcam, ab3613)	1:50
Anti-MKLP1 (Santa Cruz, sc-867)	1:1,000
Anti-pericentrin (Abcam, ab4448)	1:500
Anti-septin9	1:500
Anti-acetylated tubulin (Sigma, T7451)	1:100
Crest Serum (Antibodies Inc., 15-234)	1:50

Table S2: Primers used for real-time PCR

Gene	Right Primer	Left Primer	TM (°C)
AURKA	CCTGGCTCCCTCTGTTACAA	TTGGGTGGTCAGTACATGCT	55
AURKB	CGCCCTCCTTCTCTATCTGG	GACACCCGACATCTTAACGC	55
KIF23	CTGCCGCAATGATCCTCTTC	CGCTCTAACTCTTGCAGCAG	57
CENPE	ACCTGGCTGAGAATCCACAC	CAATGCAAGGAACGGAATTT	55
ANLN	ATCAAGGCCTGAGGGATCTT	CAGAAACCAGATGCAGCAAA	56
SEPT2	CATTCTCCACTTTCCTGCCG	AGGAAAGAAGGTCAGAGGCC	55
SEPT7	TGTTGAGCTTCCCAGTTTGC	TGGAGATGGAGCAGGTGTTT	55
TSG101	GTCACTGACCGCAGAGATGA	GATACCCTCCCAATCCCAGT	55
CHMP4	TGCTCCAGCTCCTCTAGCTC	TGCCACTACCAATGCAGAAG	55
CHMP3	CCAGAGGATCTGGGTTTGAA	AAGTCAGGAGCTGACCAGGA	55
PDCD6IP	AGGGCACGATTGATTTTGTC	TGGCTGCAAAGCACTGTATC	55
